# Loss of angiogenin function is related to earlier ALS onset and a paradoxical increase in ALS duration

**DOI:** 10.1038/s41598-020-60431-6

**Published:** 2020-02-28

**Authors:** Krishna C. Aluri, Joseph P. Salisbury, Jochen H. M. Prehn, Jeffrey N. Agar

**Affiliations:** 1 Barnett Institute of Chemical and Biological Analysis, Northeastern University, Boston, MA 02115 USA; 2 Department of Chemistry and Chemical Biology, Northeastern University, 360 Huntington Avenue, Boston, Massachusetts 02115 United States; 3 Department of Pharmaceutical Sciences, Northeastern University, 360 Huntington Avenue, Boston, Massachusetts 02115 United States; 4 Department of Physiology and Medical Physics, SFI Future-Neuro Centre, Royal College of Surgeons in Ireland, Dublin, 2 Ireland

**Keywords:** Statistical methods, Amyotrophic lateral sclerosis

## Abstract

0.5–1% of ALS (Amyotrophic Lateral Sclerosis) and Parkinson's disease (PD) are associated with mutations in the angiogenin (ANG). These mutations are thought to cause disease through a loss of ANG function, but this hypothesis has not been evaluated statistically. In addition, the potential for ANG to promote disease has not been considered. With the goal of better defining the etiology of ANG-ALS, we assembled all clinical onset and disease duration data and determined if these were correlated with biochemical properties of ANG variants. Loss of ANG stability and ribonuclease activity were found to correlate with early ALS onset, confirming an aspect of the prevailing model of ANG-ALS. Conversely, loss of ANG stability and ribonuclease activity correlated with longer survival following diagnosis, which is inconsistent with the prevailing model. These results indicate that functional ANG appears to decrease the risk of developing ALS but exacerbate ALS once in progress. These findings are rationalized in terms of studies demonstrating that distinct mechanisms contribute to ALS onset and progression and propose that ANG replacement or stabilization would benefit pre-symptomatic ANG-ALS patients. However, this study challenges the prevailing hypothesis that augmenting ANG will benefit symptomatic ANG-ALS patients. Instead, our results suggest that silencing of ANG activity may be beneficial for symptomatic ALS patients. This study will serve as a call-to-arms for neurologists to consistently publish ALS and PD patient's clinical data—if all ANG-ALS patients’ data were available our findings could be tested with considerable statistical power.

## Introduction

Amyotrophic Lateral Sclerosis (ALS) is a fatal neurodegenerative disorder characterized by the loss of upper and lower motor function and progressive muscle weakness that ultimately leads to respiratory failure and death. The overall lifetime risk of developing ALS is 1 in *ca*. 400^1^; the mean ALS onset is 60 years; and median disease duration (onset of the first symptoms until the patient's death or when respiratory assistance was required for patient's survival) reported previously ranged between 1-2 years for bulbar onset and 3-5 years for lumbar onset^2^. The most prevalent mutations associated with ALS include the genes SOD1 (superoxide dismutase 1), TARDP (TAR DNA-binding protein 43), C9orf72 (chromosome 9 open reading frame 72), ANG, FUS (RNA-binding protein FUS), Optineurin, and UBQLN2 (ubiquilin 2). There is currently no cure for ALS, though two drugs are approved for treatment. Riluzole, approved in 1995, is able to prolong survival by up to three months^3^ and Radicava (edaravone), approved in 2017, improves symptoms but has not been shown to improve survival time^4^. Since the initial identification of ANG mutations in Irish patients by Greenway *et al*.^5^, 24 mutations in ANG associated with ALS, and 12 mutations associated with Parkinson's have been reported, with these mutations being responsible for *ca*. 0.5-1% of these disorders^6,7^.

ANG is a vertebrate-specific 14.1 kDa protein belonging to the Ribonuclease A (RNase A) superfamily. ANG is expressed by a wide variety of tissues and is a secreted protein. Compared to human RNase A, human ANG has 35% sequence similarity and a significantly slower and more selective ribonuclease activity, which acts upon ribosomal and messenger RNAs (ribonucleic acid) and is required for its powerful angiogenic activity. ANG undergoes leader-sequence dependent nuclear translocation and activates transcription of ribosomal RNA^8^ through interaction with ribosomal encoding DNA containing an Angiogenin Binding Element (ABE). Its ability to induce tumor neovascularization through a potent angiogenic activity is ANG's best characterized function and provided the first major support for Folkman's tumor growth hypothesis^9,10^.

ANG performs diverse functions in addition to its angiogenic activity, often through interactions with proteins crucial to cell survival and growth. These interactions regulate cascades that mediate cell proliferation, apoptosis, and stress response, including: AKT extracellular signal-related kinase 1/2 (ERK1/2); protein kinase B/Akt; and stress-associated protein kinase/c-Jun N-terminal kinase (SAPK/JNK). ANG interaction with NF-kB results in suppression of NF-kB nuclear translocation and inflammatory response^10–13^. Upon stress in the cytoplasm ANG performs regulated cleavage of tRNA (transfer RNA) into smaller fragments termed as tiRNA, which in turn regulate protein translation and generally promote cell survival. ANG can promote neurite growth and pathfinding^14^; act as a neuroprotective and cytoprotective agent; activate microglia; and modulate astrocyte function^15,16^. The mechanisms responsible for many of ANG's non-canonical biological activities are not completely understood.

Wu *et al*. identified mutations of ANG in the American population with ALS and concluded that the loss of ribonuclease activity, nuclear translocation, or both, leads to loss of ANG function, which in turn leads to ALS^17^. Crabtree *et al*. further solidified the importance of ribonuclease activity by showing six out of seven ALS associated ANG variants studied lost ribonuclease activity^11^. Kishikawa *et al*. suggested angiogenin replacement as a potential therapy^18^. However, two variants, R121H and R121C, have 156% and 131% of WT (Wild-type) ribonuclease activity, respectively, indicating that loss of ribonuclease activity cannot be solely responsible for ANG-ALS. Previous studies used binary metrics, i.e. “yes” or “no” with respect to having ALS or RNase activity, yielding useful qualitative observations. The goal of this study is a quantitative model of ANG-ALS; in particular, how biochemical changes relate to both age-of-ALS-onset and disease duration. Such a model offers the benefit of statistically evaluating prevailing hypotheses. In addition, such a model could inform treatment strategy, by separately evaluating pre-symptomatic and symptomatic risks.

Herein, we employ a technique we term “physicochemical epidemiology,” which we developed and used to demonstrate that the length of ALS patient's disease duration (i.e. survival time after onset) depends upon the aggregation propensity and stability of fALS SOD1 (familial ALS) variants^19^. Specifically, the combined hazard ratio of SOD1 variant's loss-of-thermodynamic stability and gain-of-aggregation propensity was >300 and could account for 78% of the variability in patient's survival after onset. For comparison, the hazard ratio for smoking with respect to lung cancer is *c.a*. 12. The same study failed to identify any factor that affected the age-of-ALS-onset^19^. These findings have been corroborated in numerous studies^20–26^, and physiochemical epidemiology has since been applied to multiple diseases^25–30^. For example, ALS-associated TAR DNA-binding protein 43 (TDP-43) variants with increased stabilities (i.e. variants with longer half-lives compared to wild-type TDP-43), significantly expedite ALS onset^31^, and protein stability and activity predict the disease clinical phenotype in glucose-6-phosphate dehydrogenase deficiency^32^.

Here, using well-established epidemiological techniques, ribonuclease activity, aggregation propensity, and thermodynamic stability are assessed with respect to ALS onset and disease duration. The resulting models confirm that the loss of ANG variant's ribonuclease activity and stability contributes to ALS pathogenesis and confirm that ANG replacement is a valid pre-symptomatic treatment strategy, but contraindicate the use of ANG replacement following the clinical diagnosis of ALS symptoms.

## Results

A comprehensive literature search retrieved 13 reports, including 48 patients representing 15 ANG variants that met our inclusion criteria (see methods section for details). In addition, the Amyotrophic Lateral Sclerosis Online Genetics Database (ALSOD)^33^ and the ALS mutation database^34^ were searched, and did not produce additional data that met inclusion criteria. ALS onset data were available for all 48 ALS patients, and disease duration data were available for 30 of these 48 patients (Table 1). ANG ribonuclease activity and stability are hypothesized to affect clinical outcomes^12,17,35^ and were therefore culled from the scientific literature. Ribonuclease activity data were available for 15 variants (Table 2) and stability data were available for 7 variants (Table 3). Aggregation propensity is a significant hazard for disease duration (but not ALS onset) for patients with ALS-associated SOD1 variants. ANG variant's aggregation propensity was therefore calculated as previously described, for further evaluation with respect to ANG-associated ALS patients clinical outcomes^19^.

**Table 1:** ALS Patient onset and disease duration.

ANG Variant	Disease duration (months)	ALS Onset (years)	Reference
Q12L	>0	48	^71^
Q12L	>84	75	^71^
K17I	>0	53	^71^
K17I	>0	53	^71^
K17I	6	61	^72^
K17I	42	70	^72^
K17I	>24	72	^72^
K17I	34	68	^72^
K17I	>26	55	^72^
K17I	9	46	^73^
K17I	27	47	^74^
K17I	18	68	^74^
K17I	29	70	^69^
K17I	16	62	^69^
K17I	52	55	^69^
K17I	20	77	^69^
K17I	66	52	^75^
K17E	>36	66	^71^
K17E	9.6	83	^71^
R31K	12	66	^71^
C39W	48	45	^71^
C39W	84	47	^71^
K40I	48	45	^71^
K40I	120	27	^71^
K40I	36	70	^71^
I46V	18	76	^71^
I46V	18	41	^71^
I46V	144	45	^71^
I46V	114	46	^76^
I46V	25.2	73	^76^
I46V		54	^68^
I46V		51	^68^
I46V		45	^68^
I46V		64	^68^
I46V		55	^68^
I46V		31	^68^
I46V		60	^69^
I46V	>60	68	^77^
K54E	24	28	^78^
K54E	60	49	^79^
T80S	18	72	^69^
F100I	8	72	^69^
V103I	40	55	^80^
V113I		51	^68^
V113I		63	^68^
H114R		68	^68^
R121H	31	31	^76^
R121C*	30	72	^52^

*Concomitant G93D SOD1 mutation.

**Table 2:** Mean percent WT ribonuclease activity of ALS associated ANG variants.

ANG Variant	% Mean Ribonuclease Activity	Reference
Q12L	3.4	^11,35^
K17I	13.1	^11,35^
K17E	19.0	^11,35^
R31K	80.1	^11,35^
C39W	4.2	^11,35^
K40I	0.9	^35^
I46V	9.7	^11,35^
K54E	80.3	^11,35^
T80S	78.7	^12^
F100I	39.1	^12^
V103I	54.1	^12^
V113I	75	^35^
H114R	1.6	^12^
R121H	155.5	^35^
R121C*	131.2	^12^

*Concomitant G93D SOD1 mutation.

**Table 3:** Stability of ALS associated ANG variants.

ANG mutation	∆∆G (kcal/mol)	Reference
Q12L	−0.25	^11^
K17I	−0.48	^11^
K17E	0.15	^11^
R31K	−0.48	^11^
C39W	−5.19	^11^
K40I	−1.27	^11^
I46V	−1.50	^11^

**Figure 1:**
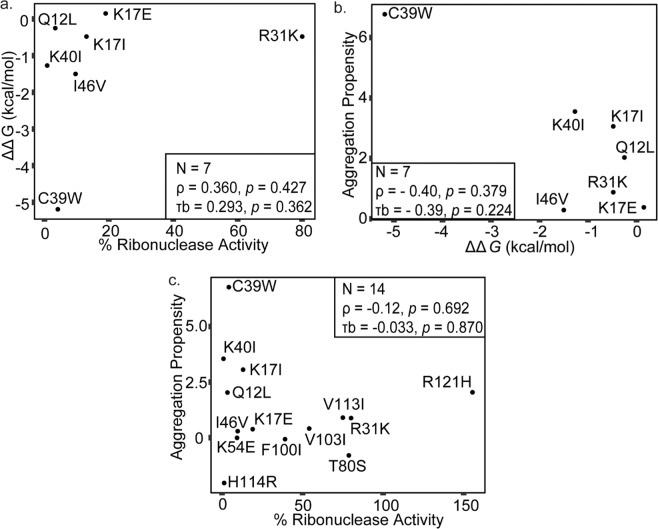
Lack of correlation between ANG stability and ANG ribonuclease activity and ANG aggregation propensity. Spearman’s Coefficient and Kendall Tau’s coefficient were used for analyzing correlation. A significance level of 0.05 was used. (**a**) Scatter plot demonstrating no significant correlation between ANG stability and ANG ribonuclease activity (**b**) Scatterplot demonstrating no significant correlation between ANG ribonuclease activity and Aggregation propensity (**c**) Scatterplot showing no significant correlation between ANG stability and aggregation propensity.

Data were tested for normalcy using Shapiro-Wilk test^36^, which indicated that ALS onset and aggregation propensity were normally distributed, but disease duration, stability and ribosomal activity were not. Non-parametric tests (Spearman's, Kendall's, Log-rank, Tarone-ware, and Breslow’s tests), one semi-parametric statistical test (Cox proportional hazards analysis), and one parametric test (Pearson’s correlation) were employed, as was appropriate (i.e. when the model’s underlying assumptions were fulfilled). To determine whether stability, ribosomal activity, and aggregation propensity of ALS-associated ANG variants were related (i.e. dependent, as might occur if loss of stability resulted in a commiserate loss of activity), their relationships were tested using Spearman’s Rho and Kendall’s Tau, which demonstrated a lack of statistical correlation between these parameters (Fig. 1). These results were consistent with: ANG stability and ribosomal activity being independent (or having a subset of components that are independent); first principles arguments and previous reports indicating lack of correlation between stability and aggregation propensity; the need to evaluate these parameters separately with respect to clinical outcomes.

Separate analyses were therefore performed with respect to a given biochemical/physicochemical characteristic (e.g. ribonuclease activity) and a given clinical outcome (e.g. ALS onset) in accordance with previously published techniques^19,20,23,24,26,32^. These analyses included the following steps: 1) correlation of a biochemical/physiochemical property and a clinical outcome (data was bootstrapped 1000 times to generate confidence intervals); 2) establishing a threshold for the biophysical/physiochemical property and separating data into two categories (e.g. categories with and without substantial ribonuclease activity); 3) performing statistical comparisons between the clinical outcomes of these categories; 4) if statistically appropriate, assigning an epidemiological hazard ratio to statistically significant categories. Specifically, the monotonic relationship between a given physicochemical parameter and clinical outcome was evaluated using Spearman’s coefficient (ρ)^37^ and Kendall’s coefficient (τ_b_)^38^. Survival data were then censored to account for patients whose ALS onset times were reported, but whose survival times were not reported; parsed according to their monotonic relationship; and Kaplan-Meier survival functions^39^ were generated. Statistical evaluations of the equality in survival functions of these categories were performed using three independent tests, which taken together, minimize potential weighting bias. These tests included Log-rank, which ranks all time points equally^40^; Tarone-ware, which is weighted by number of cases at risk at each timepoint^41^; and Breslow, which is weighted by square root of number of cases at risk^42^. Cox proportional hazards analysis was used to estimate the hazard ratio. For Cox proportional hazards analysis, the data were analyzed using two models 1) Compared the mortality risk or ALS onset risk between two categories defined above using them as categorical variables. The underlying assumptions for Cox proportionality model were evaluated graphically using log-log plots; 2) examined the association of unit increase in the physicochemical property to ALS onset risk or mortality risk using each physicochemical parameter as a continuous variable. The underlying assumptions for Cox model were evaluated using Schoenfeld residuals^43,44^.

### Loss of ANG stability correlates with faster ALS onset

**Figure 2:**
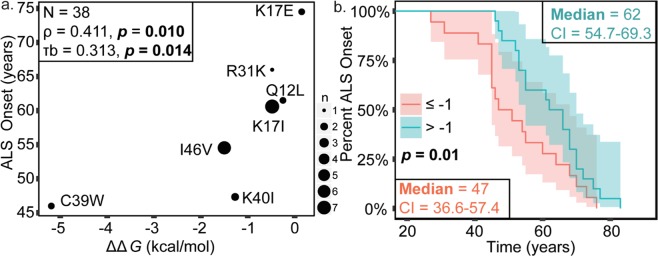
Destabilization of ANG variants correlates with faster ALS onset. Spearman’s Coefficient (*p* value 0.010) and Kendall Tau’s coefficient (*p* value 0.014) were used for analyzing correlation. Kaplan-Meier survival analysis was performed and the statistical significance of differences in survival between the categories was evaluated using Log-rank (*p* value 0.054), Breslow (*p* value 0.015), and Tarone-ware (*p* value 0.027) tests. A significance level of 0.05 was used. (**a**) Scatter plot demonstrating correlation between thermal destabilization and ALS onset. Sizes of points are proportional to number of cases. (**b**) Kaplan-Meier curves illustrating significant differences in ALS onset between patients with ANG variants with *∆∆G* less than or equal to −1 kcal/mol and variants with *∆∆G* greater than −1 kcal/mol.

To assess the relationship between ANG thermal stability and ALS onset, the stability of ANG variants (*∆∆G* = difference in variant and WT ANG ∆*G*s of unfolding) was correlated with the age of patient’s at ALS onset. A significant correlation was observed; namely, greater ANG destabilization was associated with earlier ALS onset (*p* value 0.01) (Fig. 2a, Table 4). To further investigate the relationship between ANG stability ALS onset, ANG variants were divided into two categories, variants with *∆∆G* less than or equal to −1, and variants with *∆∆G* greater than −1, and Kaplan-Meier analysis was performed (Fig. 2b). Log-rank, Tarone-Ware, and Breslow analyses of the Kaplan-Meier curves each demonstrated a significant difference between these categories (*p* values in range of 0.01–0.05) (Table 5). Specifically, patients harboring ANG variants with *∆∆G* ≤ −1 (i.e. relatively destabilized) develop ALS 15 years earlier (47 ± 5 SE (standard error) median age at onset) compared to those with relatively stable ANG variants (62 ± 4 SE median age at onset). The effect of stability on risk of ALS onset could be evaluated using Cox proportional hazards model using ∆∆*G* as a continuous variable. The overall model fit was statistically significant (p value of 0.006); indicating unit increase in stability, the risk onset decreases by 33% (hazard ratio 0.67, CI (confidence interval) 0.50–0.89). Testing for the Cox proportional hazard’s models requirements using Schoenfeld residuals (*p* value of 0.772) indicated there is no violation of the proportionality assumption.

**Table 4:** Non-parametric tests Spearman’s Rho and Kendall’s Tau to evaluate correlation of ALS onset and disease duration to physicochemical properties of ANG.

Physicochemical property		Spearman’s Rho			Kendall’s Tau			N
		Correlation coefficient	*p* value	CI	Correlation coefficient	*p* value	CI	
Stability	Ribonuclease Activity	0.360	0.427	−0.64–0.97	0.293	0.362	0.32–094	7
Stability	Aggregation Propensity	−0.396	0.379	−1.0–0.65	−0.390	0.224	−1.0–0.50	7
Ribonuclease Activity	Aggregation Propensity	−0.116	0.692	−0.68–0.60	−0.033	0.870	−0.51–0.52	14
ALS Onset	Stability	0.411	0.01*	0.10–0.67	0.313	0.014*	0.07–0.52	38
ALS Onset	Ribonuclease Activity	0.142	0.340	−0.19–0.47	0.115	0.294	−0.14–0.36	47
Disease Duration	Stability	−0.497	0.016*	−0.74–0.12	−0.385	0.02*	−0.60–0.92	23
Disease Duration	Ribonuclease Activity	−0.544	0.002*	−0.73−0.27	−0.399	0.005*	−0.58–0.19	29

*Indicates significant statistical difference.

**Table 5:** Kaplan-Meier curves’ log rank, Breslow and Tarone-ware tests are used to evaluate statistical equivalence in ALS onset, disease duration and lifespan of ANG variants with *∆∆G* less than or equal to −1 and variants with *∆∆G* greater than −1 or relative (WT) ribonuclease activity less than or equal to 10% or ribonuclease activity greater than 10%.

Physicochemical property		Log-rank		Breslow		Tarone-Ware	
		Chi-Square	*p* value	Chi-Square	*p* value	Chi-Square	*p* value
ALS Onset	Stability	3.72	0.054	5.93	0.015*	4.89	0.027*
ALS Onset	Ribonuclease Activity	1.41	0.235	2.43	0.119	1.86	0.173
Disease Duration	Stability	3.23	0.072	2.94	0.086	3.14	0.077
Disease Duration	Ribonuclease Activity	9.65	0.002*	6.72	0.01*	8.02	0.005*

*Indicates significant statistical difference.

**Figure 3:**
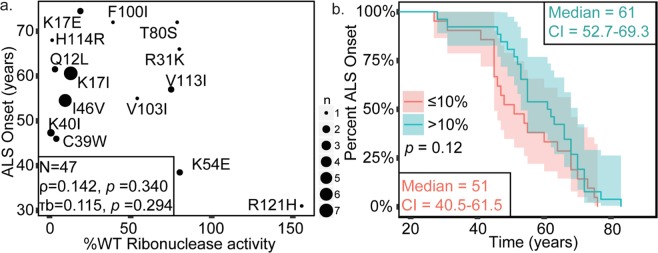
Loss of ribonuclease activity of ANG variants does not correlate with ALS onset. Spearman’s Coefficient (*p* value 0.340) and Kendall Tau’s coefficient (*p* value 0.294) were used for analyzing correlation. Kaplan-Meier survival analysis was performed and the statistical significance of differences in survival between the categories was evaluated using Log-rank (*p* value 0.235), Breslow (*p* value 0.119), and Tarone-ware (*p* value 0.173) tests. A significance level of 0.05 was used. (**a**) Scatter plot demonstrating no significant correlation between ribonuclease activity and ALS onset. Sizes of points are proportional to number of cases. (**b**) Kaplan-Meier curves illustrating no significant differences in ALS onset between patients with ANG variants with %WT ribonuclease activity less than or equal to 10% and variants with %WT ribonuclease activity greater than −10%.

To assess the relationship of ANG-ribonuclease activity and ALS onset, relative ribonuclease activities of ANG variants (to WT ANG) was correlated with age of patient’s at ALS onset. No significant correlation was observed between ALS onset and relative ribonuclease activity (*p* value range of 0.29–0.34) (Fig. 3a, Table 4). To further investigate the relationship between loss of ANG-ribonuclease activity and ALS onset, we divided the ANG variants into two categories, variants with less than or equal to ten percent WT ribonuclease activity and variants with greater than ten percnt WT ribonuclease activity and performed Kaplan-Meier analysis (Fig. 3b). The median ages of onset for patients with relatively low and high ANG activities were 51 ± 5 SE and 61 ± 4 SE years, respectively. Log Rank, Tarone-Ware, and Breslow analyses of the Kaplan-Meier curves each demonstrated no significant difference between these categories (*p* values in range of 0.12–0.24) but did trend towards the significance threshold (Table 5). The effect of ribonuclease activity on risk of ALS onset was evaluated using Cox proportional hazards model using ribonuclease activity as a continuous variable, but the overall model fit was not significant.

### Loss of ANG stability and ribonuclease activity correlate with longer ALS duration

**Figure 4:**
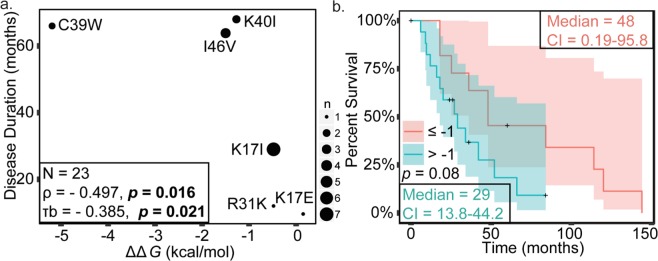
Destabilization of ANG variants correlates with longer ALS duration. Spearman’s Coefficient (*p* value 0.016) and Kendall Tau’s coefficient (*p* value 0.021) were used for analyzing correlation. Kaplan-Meier survival analysis was performed and the statistical significance of differences in survival between the categories was evaluated using Log-rank (*p* value 0.072), Breslow (*p* value 0.086), and Tarone-ware (*p* value 0.077) tests. A significance level of 0.05 was used. (**a**) Scatter plot representing correlation between thermal destabilization and disease duration. Sizes of points are proportional to number of cases. (**b**) Kaplan-Meier curves illustrating differences in ALS onset between patients with ANG variants with *∆∆G* less than or equal to −1 kcal/mol and variants with *∆∆G* greater than −1 kcal/mol.

To assess the relationship of ANG-thermal stability and ALS duration, the relative stability of ANG variants was correlated with ALS patient’s disease durations. A significant negative correlation was observed between loss of ANG stability and ALS duration (*p* value range of 0.01–0.02) (Fig. 4a, Table 4); namely, greater ANG destabilization was associated with longer ALS duration. To further investigate the relationship between stability and ALS duration, we divided the ANG variants into two categories, variants with *∆∆G* less than or equal to −1 and variants with *∆∆G* greater than −1 and performed Kaplan-Meier analysis (Fig. 4b). The median survival for patients with relatively low and high ANG activities were 48 ± 24 SE and 29 ± 8 8 SE months, respectively. Log Rank, Tarone-Ware, and Breslow analyses of the Kaplan-Meier curves each demonstrated *p* values slightly above the typical margin of significance (*p* values in range of 0.07–0.09) (Table 5). The effect of ribonuclease activity on mortality risk was evaluated by Cox proportional hazard’s analysis using ∆∆*G* as a continuous variable, but the overall model fit was not significant.

**Figure 5:**
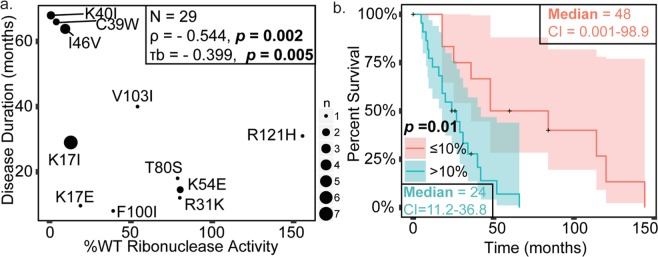
Loss of ANG ribonuclease activity correlates with longer ALS duration. Spearman’s Coefficient (*p* value 0.002) and Kendall Tau’s coefficient (*p* value 0.005) were used for analyzing correlation. Kaplan-Meier survival analysis is performed and the statistical significance of differences in survival between the categories was evaluated using Log-rank (*p* value 0.002), Breslow (*p* value 0.01), and Tarone-ware (*p* value 0.005) tests. (**a**) Scatter plot demonstrating significant correlation between ribonuclease activity and ALS onset. Sizes of points are proportional to number of cases. (**b**) Kaplan-Meier curves illustrating significant differences in disease duration between patients with ANG variants with %WT ribonuclease activity less than or equal to 10% and variants with %WT ribonuclease activity greater than −10%.

To assess the relationship between ANG ribonuclease activity and ALS duration, relative ribonuclease activity of ANG variants was correlated to disease duration of patients with ANG-associated ALS. A statistically significant negative correlation was observed between ALS duration and relative ribonuclease activity (*p* values in range of 0.002–0.005) (Fig. 5a, Table 4); namely, greater loss of ANG enzy  matic activity correlated with longer ALS duration. To further investigate the relationship between loss of ribonuclease activity and ALS duration, we divided the ANG variants into two categories, variants with less than or equal to ten percent of WT ANG ribonuclease activity and variants with greater than ten percent WT ribonuclease activity and performed Kaplan-Meier analysis (Fig. 5b; note the same results were obtained with a threshold of less than or equal to 20% ribonuclease activity). The median survival for patients with relatively low ANG activities (48 ± 26 SE) was double that of patients with relatively high ANG activities (24 ± 7 SE months). Log-rank, Tarone-Ware, and Breslow analyses each demonstrated a statistically significant difference between these categories (*p* values in range of 0.002–0.01) (Fig. 5b, Table 5). Likewise, Cox proportional hazards analysis examining mortality risk using two categories. The overall model fit was statistically significant (*p* value of 0.004) indicating ANG variants with greater than ten percent WT ribonuclease activity was associated with increased risk of death (hazard ratio = 4.1). Test for cox proportional assumptions demonstrated parallelism in log-log plot indicating no violation of proportionality hazard (Supplementary information, Figure S4). The effect of ribonuclease activity on mortality risk was evaluated using Cox proportional hazards model using WT ribonuclease activity as a continuous variable. The overall model was statistically significant (*p* value of 0.029) indicating that for every unit increase in relative percent of WT ribonuclease activity, the mortality risk increases by 1% (hazard ratio 1.01, CI 1.001–1.019, i.e. 90% increase in ribonuclease 90% increase in mortality). A test for Cox proportional hazards assumption using Schoenfeld residuals (*p* value of 0.837) indicated there is no violation of the proportionality assumption. Therefore, using a number of conservative statistical tests, loss of ribonuclease activity was significantly correlated to longer ALS duration.

Testing of additional hypotheses did not reveal any additional statistically significant relationships (Appendix S2). These included the relationship of ANG aggregation propensity to ALS clinical parameters and the relationships of ANG physicochemical parameters to ANG-related Parkinson’s clinical parameters. These results are reported in Supplementary Information (Appendix S1). Correlation of ANG nuclear translocation activity to ALS onset and survival was not performed as onset and survival data was only available for 7 patients and 5 patients, respectively with mutations showing loss of nuclear translocation ability^45^. Finally to account for multiple hypothesis testing the false discovery rate was controlled using the Benjamini-Hochberg method (Appendix S3, Table S1).

### Preclinical validation of possible deleterious effect of ANG post-ALS onset

**Figure 6:**
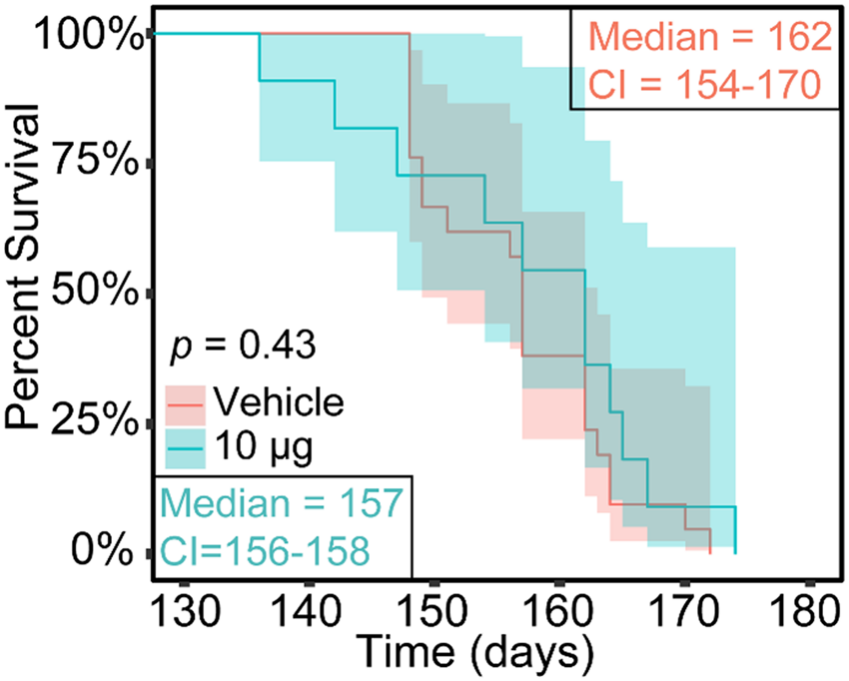
Intraperitoneal treatment of tgSOD1^G93A^-ALS mice with 10 μg recombinant huANG post disease onset did not prolong survival. (**a**) Kaplan-Meier curves illustrated no significant differences in survival between tgSOD1^G93A^-ALS mice treated with vehicle and tgSOD1G93A-ALS mice treated with 10 μg recombinant hANG (i.p., 3 times/week). Kaplan-Meier survival analysis was performed and the statistical significance of differences in survival between the categories was evaluated using Log-rank (*p* value 0.426), Breslow (*p* value 0.702), and Tarone-ware (*p* value 0.55) tests. A significance level of 0.05 was used.

We observed that in humans severe angiogenin deficiency is related to more rapid onset of ALS (10–15 years) and conversely less severe disease progression (c.a. 2 years). Taken together these suggests a protective role for presymptomatic ANG treatment and a deleterius role for postsyptomatic ANG treatment. We previously demonstrated that in SOD1^G93A^-ALS mice, a preclinical mouse model of ALS, delivery of 1 μg of human recombinant ANG intraperitoneally (3 times/week) can extend lifespan, reduce aberrant molecular expression, and improve mo tor performance^15,45^. Since our results from ALS patients indicated that higher ANG activity or stability could be deleterious post-onset, a higher 10 μg dose of ANG was administered intraperitoneally (i.p.) 3 times/week to tgSOD1^G93A^-ALS mice following the onset of ALS symptoms. Whereas 1 μg improved multiple phenotypes in tgSOD1^G93A^-ALS mice compared to vehicle-treated mice^46^, Kaplan-Meier analysis indicated that treatment with 10 μg of ANG did not (*p* value of 0.43, Fig. 6). With the caveat that the mouse line used may not be the ideal approximation of the ANG ALS population, our preclinical data provides initial evidence of enhanced ANG activity or stability indeed not been beneficial, or possibly being deleterious, post-ALS onset.

## Discussion

Correlating biochemical and molecular aspects of disease-associated variants of proteins with their respective phenotypes has aided in the understanding and diagnoses of many genetic disorders, including Huntington’s disease^47^, Alzheimer’s disease^48^, Glaucoma^49^, lysosomal storage diseases^47,50^, and ALS^19^. As previously reported^19,24^ in SOD1-associated fALS, there is a synergistic (multiplicative) correlation between variant SOD1 instability and aggregation propensity and duration of survival after ALS onset. To our knowledge, this is the first report studying the physicochemical epidemiology of ANG variants associated with ALS, as well as the first study to correlate any physical parameter to ALS onset.

Our results indicate that less stable and less active ANG variants correlate with more rapid ALS onset (15 and 10 years earlier, respectively) in humans, but paradoxically are also related to prolonged survival after onset (*c.a*. two years longer). These findings, as well as our observation of an abnormal dose-response for ANG treatment in an ALS mouse model^16,46^, have implications for how ANG replacement therapy is administered and how clinical trials for ANG replacement are designed. Clearly, if a viable ANG replacement therapy becomes available, genotyping at a young age is critical, because administering this therapy presymptomatically has the potential to increase symptom-free life by an average of 15 years. Conversely, our data raise the possibility that administering ANG replacement following ALS onset might be harmful and decrease average duration by two years. Indeed, if the data used in this study are representative of ANG variant’s biochemical and biophysical properties, and the corresponding clinical parameters of ANG ALS patient’s, ANG replacement is indicated for presymptomatic patients, and ANG gene silencing is indicated for symptomatic ALS patients. What remains unclear is whether post-symptomatic ANG toxicity is specific to ANG-ALS variants (i.e. ANG-ALS variants gain a new toxic function and wild-type ANG may still be beneficial).

How can ANG be of benefit before ALS symptoms but become harmful after ALS onset? The many beneficial roles of ANG are well-understood and led to the prevailing hypothesis that ANG replacement is warranted in ALS. Given the consensus regarding the protective roles of ANG, these are only briefly discussed here and readers are referred to the following excellent review^10^. In healthy cells ANG is known to be important for neurite growth and pathfinding^14^. As a response to cellular stress motor neurons secrete ANG, which is taken up by the astrocytes where it acts as a neuroprotective agent in a paracrine manner^14^. ANG variant K40I was also taken up by the astrocytes but lacks neuroprotective function^51^. Under stress ANG is known to trigger multiple cell signaling pathways, altering cellular transcription and cleaving tRNA to form secretory granules, and ANG knockdown using siRNA in motor neurons increased cell susceptibility to excitotoxic injury indicating neuroprotective function of ANG^16^. These are all consistent with a loss-of-ANG function being particularly detrimental to neurons that are under stress.

The observation that ANG activity and stability are related to faster ALS progression suggest a toxic role for ANG variants during the final stages of ALS. The ability of R121C to retain its ribonuclease activity and nuclear translocation ability^45^ provide initial evidence for an unknown role of ANG in ALS. A toxic role for ANG post-onset is further substantiated by clinical evidence from a patient carrying both G93D-SOD1 and ANG R121C mutations (131.2% WT ribonuclease activity). Symptoms of ALS were reported at the age of 72 years and the disease rapidly progressed for 2.5 years^52^, compared to two reported patients carrying G93D-SOD1 mutation only, previously described as slow progressing ALS mutation (Onset ranged from 45–71 years and disease progression ranged from 4 years to 22 years)^53^. Our data from tgSOD1^G93A^-ALS mice further substatiates our outcome from clincal data. The idea of beneficial entities becoming detrimental as ALS progresses is well-established. For example, the expression of Bcl2a1 is protective in G93A-SOD1-derived primary spinal cord cell cultures, until exposure to TNF-α, which mimics the condition of neuroinflammation that occurs after the onset of ALS. The authors of this study suggested that Bcl2a1 serves a protective role before onset, and promotes cell death during disease progression^54^. Similarly, such context dependant bimodal function was demostrated by NF-kB in astrocytes of tgSOD1^G93A^-ALS mice. ANG is involved in regulation of NF-κB pathway by regulating expression of four and a half LIM domains protein 3^55^. During the pre-symptomatic stage activation of NF-kB intitates microglial response showing neuroprotective role delaying onset of ALS. Whereas, post ALS onset NF-kB activation accelarates disease progression by switching the macroglial phenotype^56^. It has been shown that inhibition of NF-kB rescues motor neuron survival *in vitro* and *in vivo* ALS models^57^.

Pre-symptomatic ALS is characterized by mitochondrial deficiency, proteasome deficiency^58,59^, and motor neuron retraction^60^. ALS progression, on the other hand, is characterized by inflammation and an immune response that includes dominant astrocyte and microglial activation [51] and activation of immunoproteasomes^58,59^. Along with the role of ANG in NF-κB pathway, ANG alters the astrocyte secretome^61^ and regulates critical cellular pathways that could be either proapoptotic or antiapoptotic. ANG has been known to cause angiogenesis by activating PKB/AKT pathway^62^, activation of AKT pathway is shown to be proapoptotic by phosphorylating CDK2^63^. Nitric oxide synthesis is increased in cells by ANG^64^, nitric oxide is known for its involvement in ALS increasing cellular oxidative stress^65^. Finally, during stress ANG cleaves tRNA into fragments known as tiRNA which initiate formation of stress granules. In cells lacking NSun2, a protein that regulates tRNA methylation upon stress causes ANG induced tRNA cleavage. In this model accumulation of tiRNA was able to induce apoptosis. Further, these cells could be rescued by inhibiting ANG. ANG performs paradoxical functions depending on cell types, for example, inhibiting proliferation of hematopoietic stem/progenitor cells and enhancing proliferation in lineage-committed myeloid-restricted progenitor cells^66^.

Additional data are required to validate our physicochemical-epidemiological findings. The current data bottleneck is clinical data, which requires that more neurologist understand the importance of these data and publish them to case studies or repositories such as Amyotrophic Lateral Sclerosis Database (ALSOD)^33^ and the ALS mutation database^34^. Alternatively, if this does not happen, patient’s families should consider self-reporting these data to repositories. The major caveats of this study are: a few ANG mutants (e.g. K17I and I46V) have relatively more clinical data, leading to these mutants having a relatively large effect upon the survival analyses; and there being relatively few biochemical studies. On the other hand, biochemical studies were performed within the same labs at the same times, which bodes well for their precision. Regardless of these caveats, the whole of the biophysical and biochemical data for ANG and usable clinical data were employed here and provided consistent, statistically significant results using multiple conservative statistical approaches. These results are consistent with ALS-associated variants possessing full ANG stability and activity: prolonging lifespan by delaying onset by as much as 15 years, and paradoxically decreasing survival time after onset by as much as two years.

## Methods

Inclusion of clinical data in our study required that: 1) an ALS patient had been identified and reported in the literature to be carrying a specific ANG variant; 2) ALS-associated ANG variant had published thermal stability and/or ribonuclease activity data, and 3) individual ALS onset and/or duration of survival for that was explicitly stated that could be matched with the specific ANG variant. In order to identify publications with clinical data of patients who met our inclusion criteria, PubMed was searched as late as December 2018 for all articles mentioning a combination of either “ANG” or “angiogenin” with either “ALS” or “amyotrophic lateral sclerosis” using the string (ANG and ALS) OR (angiogenin and ALS) OR (ANG and amyotrophic lateral sclerosis) OR (angiogenin AND amyotrophic lateral sclerosis). The resulting articles were evaluated individually for ALS patient with a mutation in ANG. Publications reporting ANG mutations in ALS patients were then further evaluated to identify publications that specifically include the ALS onset data and disease duration of individual patients that had a specifically identified ANG variant that has been characterized for stability or ribonuclease activity. In extracting data from these articles, disease duration was initiated with the onset of the first symptoms until the patient’s death or when respiratory assistance was required for patient’s survival. The resulting dataset was checked against the Amyotrophic Lateral Sclerosis Database (http://www.alsod.org/)^33^ and the ALS mutation database^34^ to identify any additional data that met our inclusion criteria.

A single study obtained quantitative stability of ANG variants Q12L, K17E, K17I, R31K, C39W, K40I, and I46V (Table 3)^11^. No other stability data for ALS-associated ANG variants is available in the literature. We note that involvement of mutation K17I and I46V in ALS is supported by the majority of studies (e.g. population studies in Caucasians^67^ and Italians^68^, and molecular dynamics studies^45^) but has been questioned by a previous report^69^. Mean of ribonuclease activity was used where multiple ribonuclease activities were reported (Table 2). Aggregation propensities of mutant ANG variants was calculated from equation described by Wang *et al*^19^. Patient with the concomitant SOD1 mutation was excluded from analysis (ANG R121C). Statistical analyses were performed using R 3.4.4^70^, IBM SPSS Statistics 18 (IBM Inc., Armonk, NY, USA) and Stata SE 15 (Stata Corp LLC. College Station, TX, USA).

All animal experiments were carried out under license (AE19127/P004) from the Health Products Regulatory Authority, Ireland, with ethical approval from the Royal College of Surgeons in Ireland Research Ethics Committee (REC1122b). SOD1G93A C57B6.Cg-Tg mice (tgSOD1^G93A^-ALS mice) were purchased from Jackson Laboratory (Bar Harbor, ME, USA). Recombinant huANG (265AN250/CF, R&D Systems) stock was dissolved in sterile PBS (vehicle). SOD1G93A mice and their wild-type (WT) mice were dosed with 10 μg of huANG or vehicle via intraperitonial injection, 3 times a week from post-natal day 90 until end stage of disease^46^. Survival data of the vehicle-treated group treated alongside the 1 μg huANG group have been published previously^46^.

## Supplementary information

Supplementary Information.

## Data Availability

All data generated or analyzed during this study are included in this published article (and its Supplementary Information files).
