# Supplementary material for: Loss of angiogenin function is related to earlier ALS onset and a paradoxical increase in ALS duration
Source: Sci Rep. 2020 Feb 28;10:3715. doi: 10.1038/s41598-020-60431-6 (PMC7048737; doi:10.1038/s41598-020-60431-6)
Supplement: Supplementary file 1 — Supplementary Information. [file 41598_2020_60431_MOESM1_ESM.pdf]

Loss of angiogenin function is related to earlier ALS onset and a paradoxical increase in ALS duration.

Krishna C. Aluri[1,2], Joseph P. Salisbury[1,2], Jochen H. M. Prehn[4] and Jeffrey N. Agar[1,2,3*]

[1] Barnett Institute of Chemical and Biological Analysis, Northeastern University, Boston, MA 02115, USA.

[2] Department of Chemistry and Chemical Biology, Northeastern University, 360 Huntington Avenue, Boston, Massachusetts 02115, United States

[3] Department of Pharmaceutical Sciences, Northeastern University, 360 Huntington Avenue, Boston, Massachusetts 02115, United States

[4] Department of Physiology and Medical Physics, SFI Future-Neuro Centre, Royal College of Surgeons in Ireland, Dublin 2, Ireland

Supplementary information: Table of contents

Appendix S1: Tests for additional hypothesis

Appendix S2: Correlation of ALS lifespan with ANG physicochemical properties

Appendix S3: Adjustment of false discovery rate using the Benjamini-Hochberg method

Figure S1

Figure S2

Figure S3

Figure S4

Table S1

Appendix S1. ANG-aggregation propensity did not significantly correlate with either onset (*p* value of 0.395, CI -0.38-0.14) (Figure S1a) or ALS duration (*p* values range of 0.10-0.13, CI -1.0-0.64) (Figure S1b) or lifespan (*p* values range of 0.40-0.48, CI -0.54-0.22) (Figure S1c). Relative ANG-ribonuclease activity was correlated to the onset of PD (n = 17). No significant correlation was observed between the onset of PD and %WT ribonuclease activity of ANG variants (*p* values range of 0.59-0.60, CI -0.61-0.38) (Figure S2). Correlations with Parkinson's disease duration couldn't be performed as disease duration information was available for only two patients. Similarly, stability of ANG variants cannot be correlated with either PD onset or PD duration as $\Delta\Delta G$ data was only available for two ANG-PD variants.

Appendix S2. To illustrate the lifelong effects of ANG stability and activity, we have correlated these with the total lifespan of ALS patients by adding disease duration to ALS onset time for patients having both the data were reported (patients having only ALS onset data, but no survival data were censored). Consistent with having averaged two competing forces, no significant correlations were observed between total lifespan of ALS patients and either stability (*p* values range of 0.07- 0.08, CI -0.04-0.73) (Figure S3a) or ribonuclease activity (*p* values range of 0.56-0.73, CI -0.36-0.50) (Figure S3b). Cox proportional hazards analysis examining association of ALS lifespan using $\Delta\Delta G$ as a continuous variable, demonstrated statistical significance (*p* value of 0.009); indicating unit increase in stability, the risk mortality increases by 37% (hazard ratio 0.63, CI 0.44-0.89). Test for Cox proportional hazards assumption (*p* value of 0.568) indicated there is no violation of the proportionality assumption. Cox proportional hazards model examining association of ALS lifespan using ribonuclease activity as a continuous variable was performed. The data were not reported as the overall model fit was not significant.

Test for Cox proportional hazards assumption (*p* value of 0.293) indicated there is no violation of the proportionality assumption. Likewise, using the same thresholds to categorize ribonuclease activity and stability, Kaplan-Meier analysis performed above: the median lifespans of patients with low versus high ANG stability and ribonuclease activity were 850 ± 40.7 and 876± 148, and 876 ± 115 versus 834 ± 62.6 months, respectively; and no significant difference was observed between the survival functions of stability categories with respect to total lifespan (*p* values range of 0.18-0.28) or ribonuclease activity (*p* values range of 0.62-1) (Figures S3c and S3d). Using the same thresholds to categorize ribonuclease activity and stability, Cox proportional hazards analysis were performed: no significant difference was observed between the hazard ratio of stability categories with respect to total lifespan or ribonuclease activity categories with respect to lifespan and the data was not reported. Tests for proportionality assumption demonstrated non-parallelism between the categories indicating violation of the Cox proportionality hazard model assumption. Note, however, that patients with high ANG stability and activity survive an average of 13 and 9 years longer than the remaining ALS patients.

Appendix S3. Three covariates (ANG stability; ANG aggregation propensity; ANG ribonuclease activity) were correlated, independently, with two datasets (survival and onset). False discovery rate was therefore corrected with respect to correlating three covariates to a particular dataset. Benjamini-Hochberg method was used to adjust for the false discovery rate. A false discovery rate of 5% was used to adjust for type I error (Table S1). A total of 25 analysis were performed on our data. All our *p* values found significant in our analysis were found to be significant using Benjamini-Hochberg correction except, analysis of mortality risk using Cox proportional hazards model using WT ribonuclease activity as a continuous variable. To increase stringency, we also required significance to be achieved through multiple statistical tests of each hypothesis (e.g. correlation, Kaplan Meier, and Cox; as well as the use of survival hypothesis testing with different weighing functions using Log-rank, Tarone-ware, and Breslow).

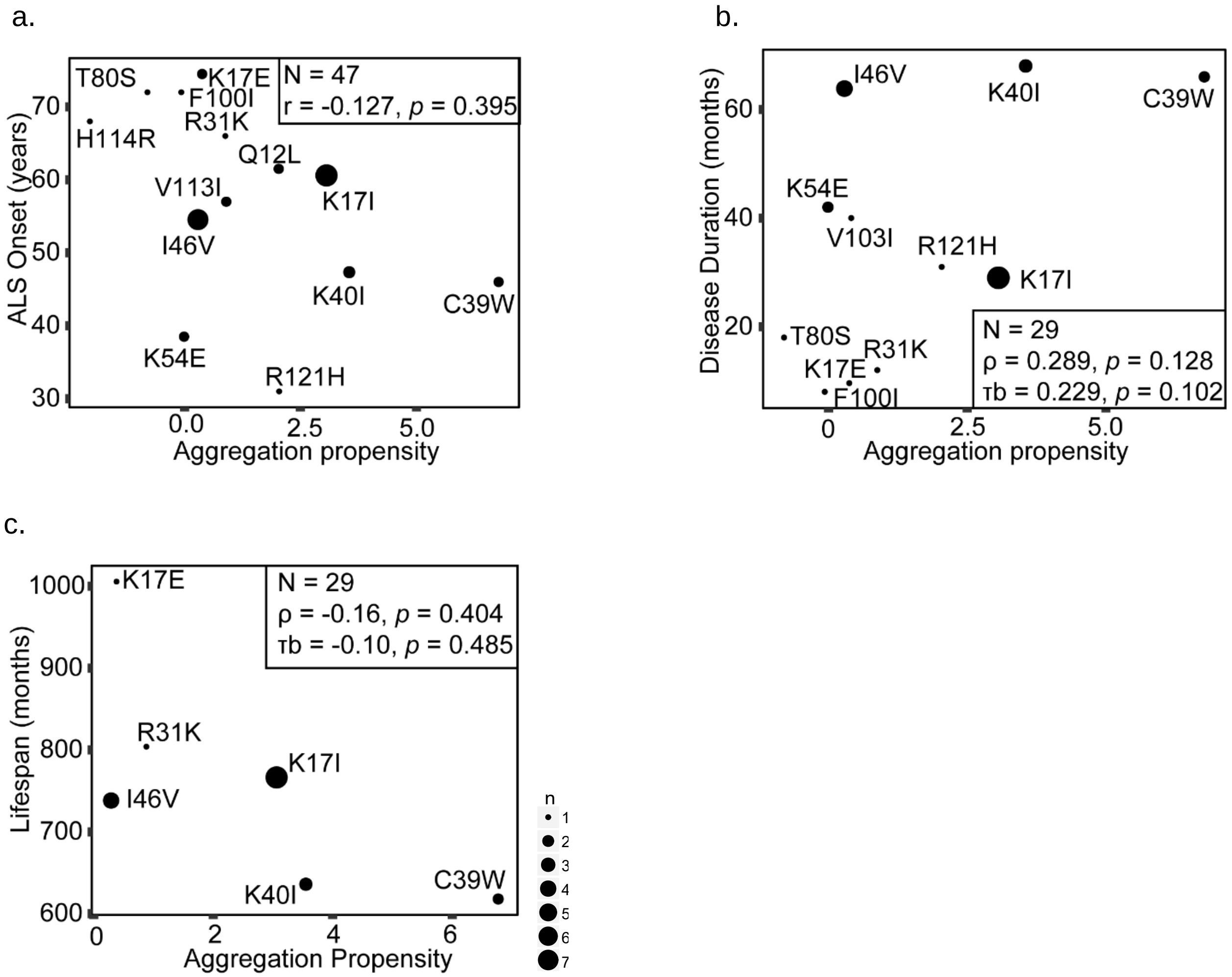

Figure S1. ALS onset, disease duration and lifespan do not correlate with aggregation propensity. a) Scatter plot demonstrating no significant correlation between aggregation propensity and ALS onset. b) Scatter plot demonstrating no significant correlation between aggregation propensity and disease duration. c) Scatter plot demonstrating no significant correlation between aggregation propensity and ALS lifespan. The definition of the aggregation propensity calculated here is important—its components (i.e. the mathematical terms used to calculate) are the fundamental physicochemical parameters of charge, hydrophobicity, and entropy. Aggregation propensity denotes the likelihood of undergoing a phase transition from an aqueous phase to an aggregated phase (i.e. it pertains to phenomena that occur following the nucleation step that "seeds" the aggregate), and to a first approximation is independent of protein stability or activity. Our study could not address the nucleation step that initiates the aggregation cascade—which could be related to protein stability—because the data were unavailable and cannot be estimated.

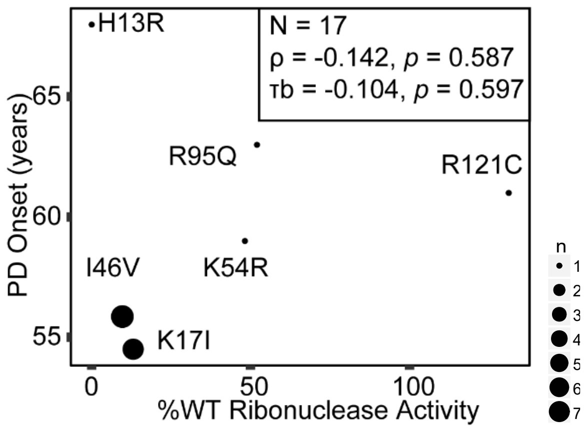

Figure S2. PD onset does not correlate with ANG ribonuclease activity. Scatter plot demonstrating no significant correlation between ribonuclease activity and PD onset.

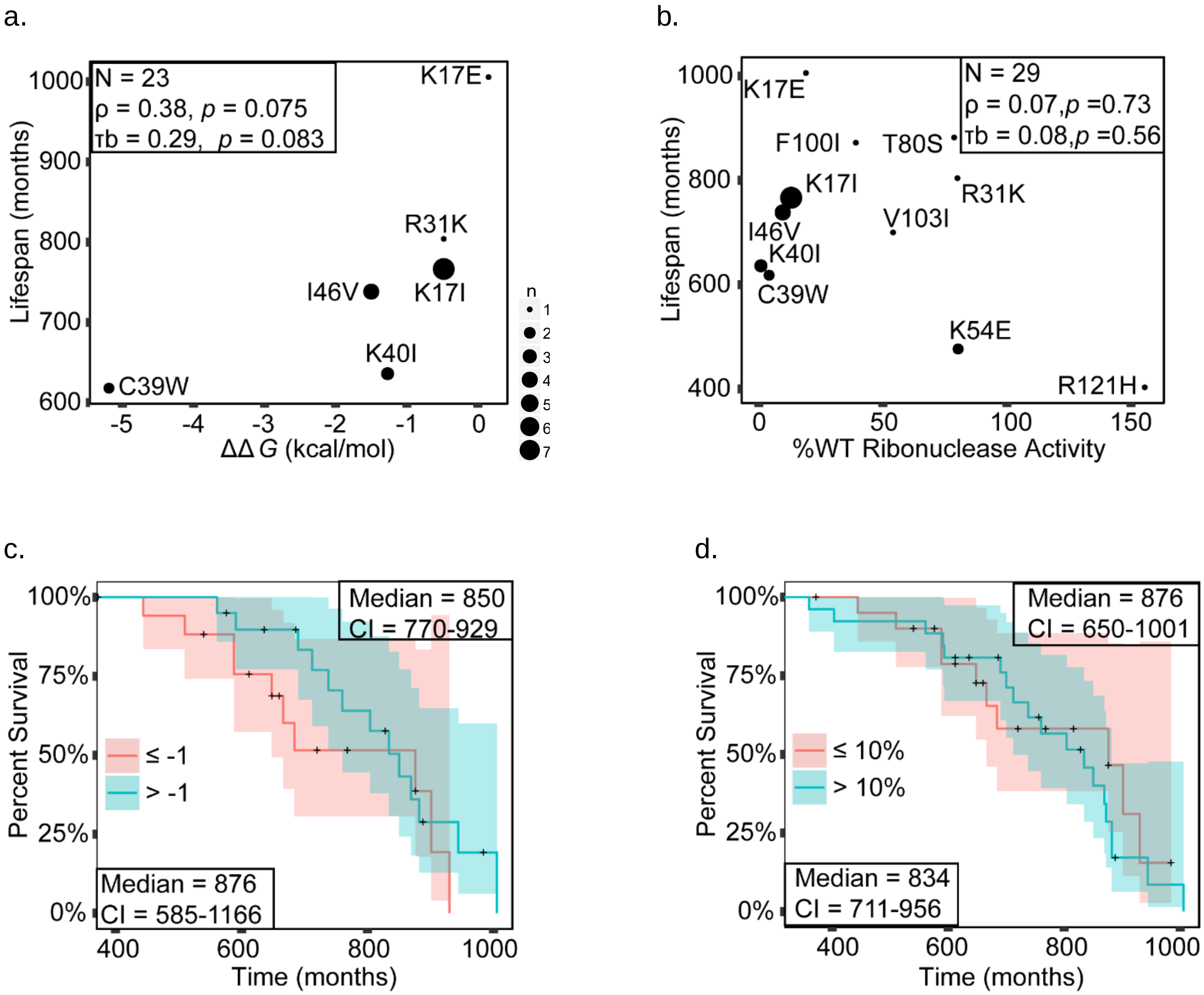

Figure S3. ALS lifespan does not correlate with stability, loss of ribonuclease activity of ANG variants. Spearman's Coefficient, Kendall Tau's coefficient were used for analyzing correlation. Kaplan-Meier survival analysis was performed and the statistical significance of differences in survival between the categories was evaluated using Log-rank, Breslow, and Tarone-ware tests. A significance level of 0.05 is used. a) Scatter plot demonstrating no significant correlation between thermal destabilization and lifespan. b) Scatter plot demonstrating no significant correlation between ribonuclease activity and lifespan. c) Kaplan-Meier curves illustrating no significant differences in lifespan between patients with ANG variants with $\Delta\Delta G$ less than or equal to -1 kcal/mol and variants with $\Delta\Delta G$ greater than -1 kcal/mol. d) Kaplan-Meier curves illustrating no significant differences in lifespan between patients with ANG variants with %WT ribonuclease activity less than or equal to 10% and variants with %WT ribonuclease activity greater than 10%.

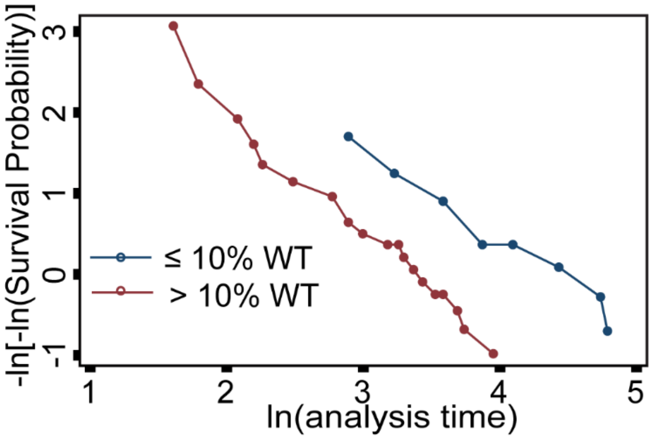

Figure S4: Test for Cox proportionality assumption. Test for proportionality was performed using log-log plot of disease duration and WT ribonuclease activity**.** Parallelism was demonstrated using log-log plots between both categories %WT ribonuclease activity less than or equal to 10% and variants with %WT ribonuclease activity greater than 10%, indicating no violation of proportionality assumption.

Table S1. Adjustment of false discovery rate using Benjamini-Hochberg method. The only *p* value lost significance upon Benjamini-Hochberg adjustment was highlighted in bold.

Physicochemical property		Type of Analysis	*p* value	Benjamini-Hochberg significance	Benjamini-Hochberg value
Disease Duration	Ribonuclease Activity	Correlation	0.002	significant	0.036
Disease Duration	Ribonuclease Activity	Cox	0.004	significant	0.036
ALS Onset	Stability	Cox	0.006	significant	0.036
Lifespan	Stability	Correlation	0.007	significant	0.036
Lifespan	Stability	Cox	0.009	significant	0.036
ALS Onset	Stability	Correlation	0.01	significant	0.036
Disease Duration	Ribonuclease Activity	Kaplan-Meier	0.01	significant	0.036
ALS Onset	Stability	Kaplan-Meier	0.015	significant	0.044
Disease Duration	Stability	Correlation	0.016	significant	0.044
**Disease Duration**	**Ribonuclease Activity**	**Cox**	**0.029**	**not significant**	**0.073**
Disease Duration	Stability	Kaplan-Meier	0.086	not significant	0.195
Disease Duration	Aggregation Propensity	Correlation	0.1	not significant	0.208
ALS Onset	Ribonuclease Activity	Kaplan-Meier	0.119	not significant	0.229

Lifespan	Stability	Kaplan-Meier	0.18	not significant	0.321
ALS Onset	Ribonuclease Activity	Correlation	0.34	not significant	0.510
ALS Onset	Ribonuclease Activity	Cox	0.343	not significant	0.510
Stability	Aggregation Propensity	Correlation	0.379	not significant	0.510
ALS Onset	Aggregation Propensity	Correlation	0.395	not significant	0.510
Lifespan	Aggregation Propensity	Correlation	0.4	not significant	0.510
Stability	Ribonuclease Activity	Correlation	0.427	not significant	0.510
Disease Duration	Stability	Cox	0.428	not significant	0.510
Lifespan	Ribonuclease Activity	Correlation	0.56	not significant	0.617
Lifespan	Ribonuclease Activity	Cox	0.568	not significant	0.617
Lifespan	Ribonuclease Activity	Kaplan-Meier	0.62	not significant	0.646
Ribonuclease Activity	Aggregation Propensity	Correlation	0.692	not significant	0.692

Table S2. Survival data of tgSOD1^{G93A}-ALS mice dosed with 10 µg ANG.

Mice ID (10ug dose)	Survival (days)
7189	136
7211	142
7187	147
7184	154
7180	157
7185	162
7186	162
7178	164
7175	165
7176	167
7182	174
